# Molecular imaging of transcriptional regulation during inflammation

**DOI:** 10.1186/1476-9255-7-20

**Published:** 2010-04-26

**Authors:** Anders Kielland, Harald Carlsen

**Affiliations:** 1 Dept. of Nutrition, Institute of Basic Medical Sciences, Faculty of Medicine, University of Oslo. PO Box 1046 Blindern, 0316 Oslo, Norway

## Abstract

Molecular imaging enables non-invasive visualization of the dynamics of molecular processes within living organisms *in vivo*. Different imaging modalities as MRI, SPECT, PET and optic imaging are used together with molecular probes specific for the biological process of interest. Molecular imaging of transcription factor activity is done in animal models and mostly in transgenic reporter mice, where the transgene essentially consists of a promoter that regulates a reporter gene. During inflammation, the transcription factor NF-κB is widely involved in orchestration and regulation of the immune system and almost all imaging studies in this field has revolved around the role and regulation of NF-κB. We here present a brief introduction to experimental use and design of transgenic reporter mice and a more extensive review of the various studies where molecular imaging of transcriptional regulation has been applied during inflammation.

## Introduction

Historically conventional imaging techniques as radiography, computed tomography, ultrasonography and magnetic resonance imaging (MRI) were developed to visualize anatomical properties and changes for diagnostic purposes. Molecular imaging, which has emerged as a new discipline during the last decade, attempts to visualize functional properties. European Society for Molecular Imaging defines molecular imaging as the characterization of the dynamics of the molecular processes in the living organisms *in vivo*. The imaging modalities in molecular imaging are SPECT and PET that detect γ- and β- radiation; MRI that detect differences in relaxation time and optical imaging that mainly record luminescent and fluorescent light [1]. Essentially there are two types of molecular methods used in imaging: 1) administration of molecular probes that recognize and bind to a particular biochemical molecule or are activated by a specific process (e.g. enzymatic reaction); 2) reporter genes that are expressed in response to a gene regulatory event. To image activation of transcription factors, most commonly genetic constructs with a promoter coupled to reporter gene are used. This requires introduction of engineered genetic constructs in research animals as in transgenic reporter mice, which has stably integrated the reporter construct in the genome.

Inflammation involves changes in hemodynamics, recruitment of leucocytes and platelets, and release of numerous signaling and effector molecules. All of this is adapted to type of tissue and stimuli (irritant, injury, infection); additionally it is timely regulated and adjusted to severity of insult. Ideally the inflammatory response is initiated on insult and terminated after homeostasis is reestablished. However, inflammation can become chronic, which is the case for diseases like rheumatoid arthritis and inflammatory bowel disease. The complexity of the inflammatory response requires that its many functional elements are controlled coordinately in some situations and independently in others. This regulation occurs through the specificity of recruited immune cells and their differentiation, signaling pathways and gene expression. Cellular protein composition is crucial for regulation at all levels, which gives transcriptional regulation a central role in orchestrating the inflammatory process. It is suggested that various sets of genes encode the different functional elements and that these genes are coordinately regulated by dedicated transcription factors [2]. For instance by using a systems biology approach in an LPS model a combination of three transcription factors (NF-κB, ATF3, CEBP/δ) was demonstrated to coordinate sustained expression of several inflammatory genes [3]. Of these, NF-κB was regarded as the activator and thus illustrates how NF-κB, which is required for most types of inflammatory responses, can engage in regulation of a specific set of inflammatory genes.

There are several hundred transcription factors involved in inflammation. In spite of this, imaging studies of NF-κB has dominated research in this field. NF-κB is attractive for inflammation studies due to the early activation, and the involvement in the large numbers of signaling pathways and the many genes related to immune functions that it controls [4,5]. The NF-κB family of transcription factors is composed of five members (p50, p52, p65, c-Rel and RelB), which can form various hetero- and homodimers. In resting cells NF-κB is retained in the cytosol bound to Inhibitors of NF-κB (IκBs). Two distinct NF-κB activation pathways have been described, the classical and the alternative pathway. In inflammation, the classical NF-κB pathway is the more important of the two, and it is activated by a large number of stimuli, including proinflammatory cytokines, bacterial and viral products, and stress-inducing stimuli such as γ-radiation, ultraviolet light and reactive oxygen species. These stimuli induce the degradation of IκBα and the nuclear translocation of mainly the p50/p65 heterodimer. In addition to being central for fighting infections and repair of tissue damage, a number of inflammatory diseases have been associated with elevated NF-κB activity including rheumatoid arthritis, inflammatory bowel disease, asthma and cardiovascular disease [6-8]. Furthermore, results from animal models with genetic manipulations that either lead to increased or decreased NF-κB activity demonstrates NF-κB's significance in regulating inflammatory pathologies [4]. Based on such findings the development of NF-κB modulators for treatment of inflammatory diseases has been given a lot of attention.

As imaging of transcription factor activity is basically only examined in transgenic reporter mice, we here briefly describe this technology. Furthermore, we review different studies related to transcription and inflammation, foremost connected to NF-κB.

## Design of transgenic reporter mice

Transgenic reporter mice have genomically inserted an engineered DNA construct (called transgene) essentially composed of a promoter and a reporter gene. Studies using reporter mice can determine activity of specific promoters and transcription factors that regulate them, which further can reflect physiological processes, disease progression and experimental manipulations. Reporter mice make possible non-invasive dynamic studies in living animals; meaning that a particular biological process can be monitored both over time and in all organs in the same animal.

**Figure 1:**
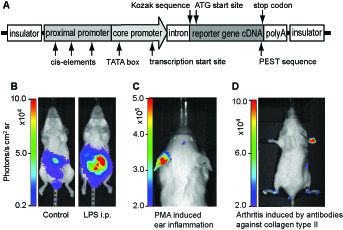
**Transgenic reporter mouse**. A) Schematic representation of a typical transgene, which is flanked by insulator sequences and including relevant elements for meaningful regulation of reporter gene expression. The core promoter often contains a TATA-box for binding of polymerase II and a transcriptional initiation site. The proximal promoter, which contains regulatory cis-elements, is usually localized upstream to the core promoter; however, enhancer elements can in principal be placed in other parts of the construct. An intron is often included to increase transcription efficiency. The polyA sequence is necessary to stabilize mRNA while the PEST sequence is introduced to exaggerate proteasome degradation and thus decrease the half-life of the protein. This is important to prevent accumulation of the reporter protein and to follow dynamic changes. Finally, the reporter gene needs the necessary elements for successful translation such as Kozak sequence and stop codon. B-D) Imaging of a transgenic reporter mouse after exposure to various inflammatory stimuli. This reporter mouse contains a transgene with NF-κB sites that regulate expression of firefly luciferase generated.

Promoters used in transgenes are in principle designed by two approaches: natural promoters taken from a gene of interest and artificial promoters where a set of selected cis-elements are combined. Often combinations of the two strategies are used. A promoter is composed of a core promoter containing DNA elements necessary for binding the polymerase and initiations of transcription, and a proximal promoter, placed upstream of the core promoter, containing regulatory cis-elements bound by transcription factors (Fig.1). In addition transgenes contain other critical elements including polyA sequences and translational start and stop codons. Furthermore, inclusion of an intron is sometimes used to increase expression efficiency [9].

Reporter genes are genetic markers that encode easily detectable proteins. The half-life of reporter proteins should be within hours when studying dynamic patterns to obtain close relationship with the biological process. Additionally, a short half-life will prevent accumulation of the reporter caused by potential background activity of the promoter. Extensive accumulation of the reporter can mask an induced expression. In basically all imaging studies of transgenic reporter mice, genes that encode bioluminescent or fluorescent proteins are used [10]. The bioluminescent reporter proteins are enzymes that catalyze a chemical reaction leading to light emission from an injected substrate. The most frequently used is firefly luciferase, but also renilla and click beetle luciferases are proven useful. Due to the scattering properties and absorption spectrum of animal tissue, reporter genes that emit light with the longest wavelength are favorable [11]. For fluorescent reporter genes this is particularly critical since autofluorescence of endogenous molecules is much lower at longer wavelengths. There are many fluorescent reporter genes available and new types are frequently introduced [12]. Recently the first infrared fluorescent protein was engineered [13]. The half-life of the fluorescent proteins are usually rather long, but the carboxy-terminal can be modified to reduce the stability of the protein [14,15]. Due to tissue autofluorescence, fluorescent imaging has much lower signal-to-noise ratio than bioluminescent imaging. However, fluorescent imaging, is superior for tomographic examination [11]. Optical imaging can be performed with a relatively rapid and easy procedure, which allows high through-put screening.

Co-regulated expression of different types of reporter genes is required to examine the same biological process with different imaging modalities. While bioluminescence is more sensitive in live animal imaging, fluorescent reporters are nearly always necessary for identification of single cells in tissue samples. In clinical imaging, expression of a therapeutic gene can be followed by co-regulated expression of a PET reporter gene [16]. To obtain co-regulated expression of genes various methods are used such as insertion of internal ribosome entry site (IRES) between the genes, repeated use of similar promoters, polyproteins, fusion proteins and bidirectional promoters. IRES is commonly used, but the expression efficiency of the genes on each side of the IRES vary according to the type of transgene and tissue [17,18]. Multiple promoters are shown to cause mutual interference, which depends on environmental factors [19]. Polyproteins are proteins intermitted with a 2A-like peptide that is "cleaved" during translation [20]. Polyproteins as well as fusion proteins require molecular engineering, which introduce sequence changes that can affect the activity, stability and immunogenicity of the proteins. Bidirectional promoters are naturally occurring in the vertebrate genome, but have not been extensively tested in transgenic models [21].

Transgenic mice are most frequently generated by pronuclear injection of fertilized eggs [22]. With this method the transgene is incorporated more or less randomly and in unpredictable copies in the genome, which create two problems. Firstly, genomic modification may unfavorably alter the phenotype of the animals because the transgene can be inserted in a location where it affects transcription. Secondly, the genomic DNA surrounding the inserted transgene can cause variable expression in different tissues through the influence of cis-elements and chromatin structure [23]. This is dependent on the location of insertion and a strategy to eliminate the problem is site specific insertion of the transgene. ROSA26 and Hprt are well described genomic loci where integration provides predictable and ubiquitous expression of inserted transgene [24,25]. This method is more laborious than pro-nuclear injection. The transgene is integrated by homologous recombination in embryonic stem cells, which are transferred to blastocysts for production of chimeric mice. A strategy to prevent influence by surrounding DNA of transgenes incorporated by pronuclear injection is the use of insulator elements. These are positioned at the ends of the transgene and have both enhancer-blocking properties preventing communications from cis-elements positioned outside the transgene, and barrier functions preventing the spread of heterochromatin [26]. As an alternative to generating transgenic reporter mice, which is time and work consuming, one can transfect liver cells by intravenous injection of a reporter construct and then study reporter activity in liver [27]. One drawback is that the expression of the reporter gene will be transient, making it difficult to perform longitudinal studies. Also viral vectors are used to visualize reporters *in vivo*, which have the potential for cell specific transfection [28]. To obtain the expected expression of reporter genes composition of the transgenic construct should be well planned with good understanding of assembled gene sequences. For instance, early versions of the firefly luciferase gene had numerous cis-elements, which took part in regulation of gene expression [29,30]. A list of unique transgenic reporter mice used to study gene regulation in inflammation is shown in table 1.

**Table 1:** Overview of transgenic reporter mice available for studies of inflammation

**Regulatory elements of transgenic mice **^**#**^	Reporter gene	Method	Features of the transgenic mice
Three NF-κB sites* separated by linker sequences (14 to 25 bp) [32,35,47,48,53,65-68]	Fluc (Firefly luciferase)	Pronuclear injection	*In vivo *imaging. Short half-life of reporter. Good induction. Used in numerous disease models. Distinct visualization of lymph nodes. Difficult to assess single cells

Three NF-κB sites* separated by linker sequences (14 to 25 bp) Insulator sequences flank the transgene [40,49,51,54,69-76]	Fluc	Pronuclear injection	*In vivo *imaging. Short half-life of reporter. Insulators protect transgene against genomic interference. Used in many disease models. Difficult to assess single cells.

Six NF-κB sites* separated by four bp. Bi-directional expression of two reporter genes [77]	Fluc dEGFP	Pronuclear injection	*In vivo *imaging and detection of dEGFP in single cells. Short half-life of both reporters. Used in a brain ischemia model. Weak dEGFP signal. Need antibodies for detection.

HIV-1 LTR with two NF-κB sites* and three Sp1 sites [31,36,38,41,52,78-82]	Fluc	Pronuclear injection	*In vivo *imaging. Short half-life of reporter. Good induction. Used in various disease models mainly to study lung pathology. Difficult to assess single cells.

HIV-1 LTR with two NF-κB sites* and three Sp1 sites [37]	EGFP/Fluc fusion protein	Pronuclear injection	*In vivo *imaging. EGFP signal detected in isolated macrophages. Short half-life of both reporters. Good induction. Need antibodies to detect EGFP in sections.

Two NF-κB sites* [83-86]	Fluc	Pronuclear injection	Good induction. Successfully used to study T-cell regulation. No demonstration of *in vivo *imaging.

Five NF-κB sites *[87]	Fluc	Pronuclear injection	*In vivo *imaging. Short half-life of reporter. Used in only one study.

Three NF-κB sites* [88]	EGFP	Site specific in HPRT-locus	Signals detected from single cells and whole organs. Site specific integration prevents influence from regulatory elements outside the transgene. *In vivo *imaging not shown. Stable version of EGFP complicates assessment of dynamic NF-κB regulation.

Twelve Smad 2/3 binding sites [55,89,90].	Fluc	Pronuclear injection	*In vivo *imaging. Used to study TGFβ signaling and response to injury, particularly in brain. Difficult to assess single cells.

iNOS-promoter fragment (1.24 kb) [57]	Fluc	Pronuclear injection	*In vivo *imaging. Reflects iNOS mRNA in liver. Sensitive to pro- and anti-inflammatory agents. Used in only one study.

IκBα-promoter fragment (11.0 kb) [58]	Fluc	Pronuclear injection	*In vivo *imaging. Luciferase activity reflects IκBα mRNA in liver. Used in only one study.

SAA1-promoter fragment (7.7 kb) [59]	Fluc	Pronuclear injection	*In vivo *imaging. Luciferase activity reflects SAA1 mRNA in liver and protein in serum. Demonstrated in an acute arthritis model. Used in only one study.

GADD45β-promoter fragment (10.5 kb) [60]	Fluc	Pronuclear injection	*In vivo *imaging. Reflects GADD45β mRNA in multiple organs. Used to study effects of various stressful insults (inflammation, oxidative stress, toxins). Used in one study.

COX-2-promoter (endogenous) [91-93]	Fluc	Knock-in in the COX2 gene	*In vivo *imaging. Correlation between luciferase and COX-2 protein levels in multiple organs. Knock-in reflects endogenous promoter activity.

^#^Include most relevant references where the models have been used.

*Sequence of the NF-κB binding site: 5'-GGGACTTTCC-3'. This sequence is found in numerous NF-κB regulated promoters including immunoglobulin κ light chain and HIV LTR, and it is used in all NF-κB reporter mice generated up to now.

## Applications of NF-κB transgenic reporter models

The two first transgenic mice that convincingly showed that NF-κB activity could be imaged *in vivo *either used the HIV long terminal repeat as promoter [31] or a synthetic promoter containing three NF-κB binding sites from the immunoglobulin κ light chain promoter [32]. Both mice utilized the luciferase from firefly as reporter gene to mediate light emission. Activation of NF-κB by classical stimuli (LPS, IL-1β and TNFα) induced the expression of luciferase, which could be followed in the same animal over time and in multiple organs. Increase in NF-κB activity during development of arthritis was visualized, as well as decrease by the anti-inflammatory agent Dexamethasone. Furthermore, NF-κB dependent luciferase activity of individual organs was imaged after dissection from sacrificed animals and the signal strength in these *ex vivo *images was equal to the luciferase activity recorded in tissue homogenates. This confirms that the luminescent signal recorded in tissue reflects the actual level of reporter protein. These reporter mice have been used to examine the role of NF-κB during inflammation, inflammatory mechanisms in general and to evaluate therapeutic strategies. A selection of these studies is reviewed in this chapter with a summary listed in table 2.

**Table 2:** Overview of imaging studies related to transcriptional regulation in inflammation

Type of study	Results
Imaging neural regulation of NF-κB	Hepatic NF-κB is crucial for recruitment of neutrophils to the injured brain [33]. Vagus nerve signaling regulates NF-κB activity [35].

Imaging of infection models	*In vivo *imaging of NF-κB in lung [31]. NF-κB is sufficient to cause lung inflammation [36]. Duration of NF-κB activity is determining for lung injury [37]. Bacterial lung infection induces NF-κB. Lack of oxidative burst and targeted inhibition of NF-κB worsens Pseudomonas infection [38,39]. NF-κB is induced in infected mammary glands [40]. NF-κB is central for regulating milk production of mammary glands [41].

Imaging autoimmune disease	*In vivo *imaging of NF-κB during arthritis [32]. Tracking NF-κB in a transgenic model with various autoimmune diseases [47]. Evaluation of NF-κB inhibitor in arthritis. Combined imaging of NF-κB activity and protease specific near infrared probe [48]. Evaluation of probe for reactive oxygen species and NF-κB activity in arthritis [49].

Imaging of dietary influence	NF-κB activity during high fat feeding and obesity [51]. NF-κB and its role in energy balance of obese mice [52]. Vitamin A regulates NF-κB activity [54].

Imaging host immune reaction	Interaction between host and biomaterial induces NF-κB [87]

Imaging TGFβ signaling	Imaging Smad2/3-dependent TGF-beta signaling reveals prominent tissue-specific responses to inflammatory stimulus and injury [55]. Orally administered TGF-beta is biologically active in the intestinal mucosa and enhances oral tolerance [89]. Imaging of Smad signaling shows correlation with excitotoxic neurodegeneration [90].

Imaging regulation through natural promoters of inflammatory genes	iNOS-promoter activity used to evaluate effect of anti-inflammatory compounds [57]. Regulation of IκBα expression involves both the NF-κB and MAP kinase signaling pathways [58]. Serum amyloid A is induced by inflammatory stimuli. NF-κB is an important regulator [59]. GADD45β-promoter regulation by NF-κB and not MAPK pathway in acute inflammation [60]. Imaging Cox-2 gene expression in living animals with a luciferase knock-in reporter gene [93].

Imaging inflammation in non-conventional transgenic mice	NF-κB activation during liver inflammation in mice and prevention by catalase delivery [61]. Real-time imaging of ligand-induced IKK activation in liver [62]. Viral delivery of reporter constructs to discrete brain region used to monitor longitudinal NF-κB and AP1 activity [63].

### Imaging neural regulation of NF-κB

Previous observations have shown that following acute brain injury, leukocytes are recruited particularly from the liver to the damaged brain. To test the hypothesis that NF-κB has a critical role in this process, the dynamics of NF-κB activity was imaged after induction of brain injury by intracerebral injection of IL-1β. This led to an exceptionally rapid NF-κB activation in the liver, suggestive of a signal transfer that involves the neural system. To determine the role of hepatic NF-κB, it was selectively inhibited by intravenous adenoviral-mediated delivery of an IκBα super-repressor. This treatment significantly reduced the number of neutrophils recruited to the brain [33].

The vagus nerve is shown to stimulate anti-inflammatory processes in the gut through cholinergic modulation of macrophages [34], and *in vivo *imaging of the gut region after blocking signaling from the vagus nerve by transection showed increased NF-κB activity. Furthermore, in mice with experimentally induced colitis cutting the vagus nerve, and thus removing the cholinergic mediated anti-inflammatory signal, exaggerated the NF-κB activity. This elevated NF-κB activity coincided with disease severity and reduction in regulatory T-cells [35].

### NF-κB imaging in models of infectional diseases

Various constitutively active forms of IκB kinases have been virally introduced in airway epithelium to test whether activation of NF-κB pathways are sufficient to generate lung inflammation [36]. *In vivo *imaging was used to confirm up-regulation of NF-κB activity in the lung, which also correlated well with disease parameters as cytokines, chemokines and recruitment of neutrophils. Imaging was also used to investigate to what extent duration of NF-κB activation correlated with outcome of lung inflammation [37]. Models of acute or chronic lung infection were induced in reporter mice with single injection or continuous administration of LPS, respectively. NF-κB activation was stronger and more sustained in mice with chronic disease, which progressed into more severe lung injury. Furthermore, an NF-κB inhibitor (BMS-345541), which was delivered after onset of inflammation, reduced disease severity in parallel with reduction in NF-κB activity. Although NF-κB activity clearly correlated with disease outcome in the LPS model described above, two other studies shows that the host defense against Pseudomonas bacterial infection is impaired when NF-κB activity is inhibited experimentally [38,39].

Mastitis is defined as inflammation of the mammary gland mainly caused by microbial pathogens, such as bacteria. Mastitis is quite common in breast feeding women, and in the dairy industry intra-mammary infections are of great economical importance due to loss of milk production. The dynamics of NF-κB activity was investigated in a mouse model of mastitis where E.coli was inoculated in the mammary glands of lactating mice [40]. NF-κB was rapidly, but transiently activated, with a peak around 10 hours and termination after 24 hours. Interestingly, a systemic response was revealed as a mild increase in NF-κB activity in the liver, which also was longer lasting. This systemic reaction was confirmed by increased circulating levels of the acute phase protein serum amyloid A, tumour necrosis factor-α and interleukin-6. Interestingly, in a recent work it was shown that activation of NF-κB in mammary glands was sufficient to cause mastitis-like symptoms such as increased apoptosis and loss of milk production. Oppositely, specific inhibition of NF-κB in glands of mice with mastitis prevented milk loss [41]. These results indicate that NF-κB is a critical regulator of milk loss during infection, making NF-κB reporter mice useful to evaluate therapeutic strategies.

### NF-κB imaging in autoimmune disease

Despite intense research efforts, the etiology of most autoimmune diseases remains obscure. It is previously shown that B-cells can present fragments of the variable region of their immunoglobulins, called idiotype (Id), on their MHC class II, which further can be recognized by T-helper cells. Such T-cells with Id-specific receptors have been described in a number of autoimmune diseases in humans [42-45]. In a mouse model where the collaboration between Id-presenting B-cell and Id-specific T cell are enhanced through genetic manipulation, a plethora of autoimmune diseases correlating with autoantibody production develops [46]. To determine the role of NF-κB during initiation and progression of autoimmune diseases, the mouse model was crossed with NF-κB reporter mice. Imaging revealed NF-κB activation before onset of clinical symptoms and it correlated with disease progression and autoantibody production. Activation was observed in secondary lymphoid organs, inflamed colon, skin lesions, and arthritic joints. Moreover, *ex vivo *imaging of the small intestine demonstrated autoimmune disease, which had clinical parameters in agreement with celiaki [47]. Additionally, imaging of NF-κB activation has been used to quantify the effect of the IκB kinase 2 inhibitor ML120B in a model of rheumatoid arthritis. This was verified by reduced expression of NF-κB target genes [48]. These results suggest that *in vivo *imaging of NF-κB activation is a good marker for autoimmune disease in experimental mouse models.

### Imaging of NF-κB together with new optical probes

Numerous molecular processes are involved in inflammation and probes that can detect some of these events have been developed. Imaging of NF-κB activation together with such probes has been performed to examine correlation of different but still connected processes. A near infrared fluorescent probe that emits light when cleaved by the protease activity of cathepsin B and K was utilized in a model of rheumatoid arthritis [48]. The intensity of the probe coincided with disease severity and NF-κB mediated luminescence intensity. Production of reactive oxygen species is a hallmark of inflammation. A bioluminescent probe (L-012) that reacts with some of these reactive oxygen species was shown to be activated in parallel with NF-κB in various inflammation models [49].

### NF-κB imaging of dietary influence

The discovery that diet affects gene regulation has been crucial for the understanding of diet's role in health and disease. It has been demonstrated that dietary components can be both pro-inflammatory and anti-inflammatory. Intake of high fat diet can for instance lead to low grade inflammation, which again is linked to metabolic syndrome and type 2 diabetes [50]. In two related studies, NF-κB reporter mice were fed high fat diet for several weeks and both studies found a modest but significant increase in NF-κB dependent bioluminescence, indicative of low grade inflammation [51,52]. Interestingly, in one of these studies the NF-κB target gene IκBε was chronically elevated, whereas genetic manipulations to inhibit this gene protected against type 2 diabetes [52]. Diet can also contribute to a reduction in inflammation through regulation of NF-κB. Previous studies showed that vitamin A deficiency was linked to increased infection and inflammation. In an attempt to clarify the relationship of vitamin A and NF-κB activity, vitamin A deficient diet were fed to NF-κB reporter mice to deplete the vitamin A stores. Vitamin A deficient mice had an overall increased NF-κB induction of 2.2 fold. Conversely, when mice on normal diet were given a single oral dose of vitamin A in the form of retinoic acid, NF-κB activity was rapidly and transiently decreased [53]. This inhibition was also observed in LPS treated mice [54]. Thus, the use of NF-κB reporting mice may prove to be a powerful tool to evaluate anti-inflammatory effects of other dietary factors.

## Imaging TGFβ signaling

Besides NF-κB reporter mice, very few other transgenic reporter models exists for reporting specific transcription factors related to inflammation. One exception is the luciferase reporter mice for the transcription factors Smad 2 and 3 [55]. Activation of these transcription factors is the canonical signaling pathway for transforming growth factor β (TGFβ). TGFβplays a wide role in the immune system, and affects all populations of leukocytes in a stimulatory or inhibitory manner [56]. The Smad reporter mouse was used to assess global regulation of Smad 2 and 3 activities after LPS stimulation. LPS rapidly induced luciferase expression in liver and brain. Moreover, the signal was much more prolonged in the brain demonstrating differences in organ regulation by TGFβ signaling.

## Imaging promoter regulation relevant for inflammation

In addition to imaging inflammatory regulation of single transcription factors, several reporter mice have been developed for studies of natural promoters involved in inflammation. Such reporter mice are valuable both for assessing the transcriptional regulation during inflammation and to evaluate the relative contribution of distinct inflammatory genes. Zhang et al., have developed four different transgenic luciferase reporter mice with promoters from important response genes in inflammation: Serum Amyloid A (SAA), inducible Nitric Oxide Synthetase (iNOS), IκB and Growth arrest and DNA-damage-inducible β (GADD45β). *In vivo *imaging following induction of inflammation by various stimuli showed robust activation in all the reporter mice. In addition they showed, by exploiting specific inhibitors and activators, that NF-κB is central to the regulation of all the promoters. However, the influence of NF-κB differed between the four. The SAA and GADD45β promoter were primarily regulated by NF-κB, whereas the IκB promoter was in addition influenced by the p38 signaling pathway and the iNOS promoter also needed interferon regulated factor for maximal activation [57-60].

## Imaging non-conventional transgenic mice

While basically all studies of molecular imaging of transcriptional regulation in inflammations is done in mice produced by pronuclear injection there are some studies with other methods. For instance, transfection and expression of reporter genes can specifically be obtained in liver following intravenous injection of naked DNA. Such an approach has been used to image luciferase activity under the control of NF-κB [61]. In these mice, administration of thioacetamide or LPS showed strong induction of liver bioluminescence and the signal was reduced by catalase. Using the same type of method a fusion protein between firefly luciferase and IκBα was expressed in liver cells. Degradation of IκB is prerequisite for activation of NF-κB. Since degradation of IκB also leads to degradation of firefly luciferase, disappearance of light emission will reflect NF-κB activation [62]. This approach enables a close to real-time imaging of the NF-κB signaling pathway. Another approach is to utilize adenoviral transfection. Brain nuclei have been transfected with reporter constructs for NF-κB and AP1 and imaged *in vivo*. In this study, luciferase activity could be quantified and followed over several weeks after LPS stimulation [63].

## Conclusion and future perspective

Molecular imaging of transcription factors is a young research field, but has demonstrated the usefulness of visualizing regulation of transcription factors *in vivo*. We have basically only reviewed studies on NF-κB regulation in inflammation, which have dominated this research area. The various studies illustrate the importance and advantage both to track activity in individual mice over time, to quantify the relative changes in activity and to visualize the spatial patterns of activation.

The present reporter models of NF-κB have provided valuable information in a variety of experiments, but still have potential to elucidate a number of unresolved questions related to inflammation. However, NF-κB, whose binding sites are represented in more than 200 genes, is a rather ubiquitous marker of inflammation and it is also present in genes not directly involved in inflammation. Therefore, development of promoters that are more specifically regulated is attractive in order to investigate distinct functional elements of inflammation. Such promoters must be under regulation of defined sets of transcription factors. An important improvement in this regard will be the ability to more precisely image gene regulation in individual organs, and functional events such as activation of specific cell types and productions of distinct cytokines.

Due to the rapid evolution of imaging technology combined with advances in molecular biology more quantitative and localized characterization of the reporter signal will represent an important and valuable improvement. The studies presented in this review have utilized conventional two dimensional imaging of reporter gene activity, which indeed has created valuable information from distinct organs such as liver, lungs, brain and intestine; however, due to light scattering and poor tissues penetration, it is difficult to localize and quantify signals from deeper lying tissues. Information on inflammation in specific organs is vital for the understanding of biological mechanisms and to validate more precisely the effect of a treatment regime. New developments of brighter versions of optical reporter proteins as well as more red shifted fluorescent proteins is therefore crucial for obtaining tissue specific imaging. The combination of anatomical images (Computer tomography and MRI) with molecular imaging is forthcoming [64] and will clearly be utilized more extensively in anatomical characterizations. Molecular imaging of transcription factor activity in inflammation will definitively also in the future be an essential tool to provide knowledge in basic biological mechanisms in preclinical studies.

## Competing interests

HC owns stocks in the company Cgene AS, who holds the commercial rights of certain NF-κB-luciferase reporter mice. AK has been partly employed by Cgene AS.

## Authors' contributions

AK and HC authored the manuscript. Both authors read and approved the final manuscript.

## Acknowledgements

This work was funded by grants from the EU consortium DiMI (LSHB-CT-2005-512146) and the Norwegian Research Council.

## References

[B1] Kang JH Chung JK Molecular-genetic imaging based on reporter gene expression J Nucl Med 2008 49 Suppl 2 164S 179S 10.2967/jnumed.107.045955 18523072

[B2] Medzhitov R Horng T Transcriptional control of the inflammatory response Nat Rev Immunol 2009 9 692 703 10.1038/nri2634 19859064

[B3] Litvak V Ramsey SA Rust AG Zak DE Kennedy KA Lampano AE Nykter M Shmulevich I Aderem A Function of C/EBPdelta in a regulatory circuit that discriminates between transient and persistent TLR4-induced signals Nat Immunol 2009 10 437 443 10.1038/ni.1721 19270711 PMC2780024

[B4] Pasparakis M Regulation of tissue homeostasis by NF-kappaB signalling: implications for inflammatory diseases Nat Rev Immunol 2009 9 778 788 10.1038/nri2655 19855404

[B5] Vallabhapurapu S Karin M Regulation and function of NF-kappaB transcription factors in the immune system Annu Rev Immunol 2009 27 693 733 10.1146/annurev.immunol.021908.132641 19302050

[B6] Feldmann M Brennan FM Maini RN Rheumatoid arthritis Cell 1996 85 307 310 10.1016/S0092-8674(00)81109-5 8616886

[B7] Barnes PJ Adcock IM Transcription factors and asthma Eur Respir J 1998 12 221 234 10.1183/09031936.98.12010221 9701442

[B8] Neurath MF Becker C Barbulescu K Role of NF-kappaB in immune and inflammatory responses in the gut Gut 1998 43 856 860 9824616 10.1136/gut.43.6.856 PMC1727350

[B9] Brinster RL Allen JM Behringer RR Gelinas RE Palmiter RD Introns increase transcriptional efficiency in transgenic mice Proc Natl Acad Sci USA 1988 85 836 840 10.1073/pnas.85.3.836 3422466 PMC279650

[B10] Gross S Piwnica-Worms D Spying on cancer: molecular imaging in vivo with genetically encoded reporters Cancer Cell 2005 7 5 15 15652745 10.1016/j.ccr.2004.12.011

[B11] Weissleder R Ntziachristos V Shedding light onto live molecular targets Nat Med 2003 9 123 128 10.1038/nm0103-123 12514725

[B12] Shcherbo D Murphy CS Ermakova GV Solovieva EA Chepurnykh TV Shcheglov AS Verkhusha VV Pletnev VZ Hazelwood KL Roche PM Far-red fluorescent tags for protein imaging in living tissues Biochem J 2009 418 567 574 10.1042/BJ20081949 19143658 PMC2893397

[B13] Shu X Royant A Lin MZ Aguilera TA Lev-Ram V Steinbach PA Tsien RY Mammalian expression of infrared fluorescent proteins engineered from a bacterial phytochrome Science 2009 324 804 807 10.1126/science.1168683 19423828 PMC2763207

[B14] Li X Zhao X Fang Y Jiang X Duong T Fan C Huang CC Kain SR Generation of destabilized green fluorescent protein as a transcription reporter J Biol Chem 1998 273 34970 34975 10.1074/jbc.273.52.34970 9857028

[B15] Corish P Tyler-Smith C Attenuation of green fluorescent protein half-life in mammalian cells Protein Eng 1999 12 1035 1040 10.1093/protein/12.12.1035 10611396

[B16] Jacobs A Voges J Reszka R Lercher M Gossmann A Kracht L Kaestle C Wagner R Wienhard K Heiss WD Positron-emission tomography of vector-mediated gene expression in gene therapy for gliomas Lancet 2001 358 727 729 10.1016/S0140-6736(01)05904-9 11551583

[B17] Borman AM Le Mercier P Girard M Kean KM Comparison of picornaviral IRES-driven internal initiation of translation in cultured cells of different origins Nucleic Acids Res 1997 25 925 932 10.1093/nar/25.5.925 9023100 PMC146526

[B18] Hennecke M Kwissa M Metzger K Oumard A Kroger A Schirmbeck R Reimann J Hauser H Composition and arrangement of genes define the strength of IRES-driven translation in bicistronic mRNAs Nucleic Acids Res 2001 29 3327 3334 10.1093/nar/29.16.3327 11504870 PMC55851

[B19] Emerman M Temin HM Genes with promoters in retrovirus vectors can be independently suppressed by an epigenetic mechanism Cell 1984 39 449 467 10.1016/0092-8674(84)90453-7 6096005

[B20] Szymczak AL Vignali DA Development of 2A peptide-based strategies in the design of multicistronic vectors Expert Opin Biol Ther 2005 5 627 638 10.1517/14712598.5.5.627 15934839

[B21] Amendola M Venneri MA Biffi A Vigna E Naldini L Coordinate dual-gene transgenesis by lentiviral vectors carrying synthetic bidirectional promoters Nat Biotechnol 2005 23 108 116 10.1038/nbt1049 15619618

[B22] Hogan B Beddington R Constantini F Lacy E Manipulating the mouse embryo Cold Spring Harbor Laboratory Press 1994

[B23] Wilson C Bellen HJ Gehring WJ Position effects on eukaryotic gene expression Annu Rev Cell Biol 1990 6 679 714 10.1146/annurev.cb.06.110190.003335 2275824

[B24] Awatramani R Soriano P Mai JJ Dymecki S An Flp indicator mouse expressing alkaline phosphatase from the ROSA26 locus Nat Genet 2001 29 257 259 10.1038/ng1101-257 11687793

[B25] Bronson SK Plaehn EG Kluckman KD Hagaman JR Maeda N Smithies O Single-copy transgenic mice with chosen-site integration Proc Natl Acad Sci USA 1996 93 9067 9072 10.1073/pnas.93.17.9067 8799155 PMC38596

[B26] Gaszner M Felsenfeld G Insulators: exploiting transcriptional and epigenetic mechanisms Nat Rev Genet 2006 7 703 713 10.1038/nrg1925 16909129

[B27] Liu F Song Y Liu D Hydrodynamics-based transfection in animals by systemic administration of plasmid DNA Gene Ther 1999 6 1258 1266 10.1038/sj.gt.3300947 10455434

[B28] Davidson BL Breakefield XO Viral vectors for gene delivery to the nervous system Nat Rev Neurosci 2003 4 353 364 10.1038/nrn1104 12728263

[B29] Prathalingam SR Howard A Barley NF Legon S Walters JR Inhibition of luciferase expression from a commercial reporter vector by 1,25-dihydroxycholecalciferol Anal Biochem 1998 263 113 115 10.1006/abio.1998.2815 9750151

[B30] Kogai T Kanamoto Y Brent GA The modified firefly luciferase reporter gene (luc+) but not Renilla luciferase is induced by all-trans retinoic acid in MCF-7 breast cancer cells Breast Cancer Res Treat 2003 78 119 126 10.1023/A:1022179717847 12611464

[B31] Sadikot RT Jansen ED Blackwell TR Zoia O Yull F Christman JW Blackwell TS High-dose dexamethasone accentuates nuclear factor-kappa b activation in endotoxin-treated mice Am J Respir Crit Care Med 2001 164 873 878 11549548 10.1164/ajrccm.164.5.2008059

[B32] Carlsen H Moskaug JO Fromm SH Blomhoff R In vivo imaging of NF-kappa B activity J Immunol 2002 168 1441 1446 11801687 10.4049/jimmunol.168.3.1441

[B33] Campbell SJ Anthony DC Oakley F Carlsen H Elsharkawy AM Blomhoff R Mann DA Hepatic nuclear factor kappa B regulates neutrophil recruitment to the injured brain J Neuropathol Exp Neurol 2008 67 223 230 10.1097/NEN.0b013e3181654957 18344913

[B34] Wang H Yu M Ochani M Amella CA Tanovic M Susarla S Li JH Yang H Ulloa L Al-Abed Y Nicotinic acetylcholine receptor alpha7 subunit is an essential regulator of inflammation Nature 2003 421 384 388 10.1038/nature01339 12508119

[B35] O'Mahony C Kleij H van der Bienenstock J Shanahan F O'Mahony L Loss of vagal anti-inflammatory effect: in vivo visualization and adoptive transfer Am J Physiol Regul Integr Comp Physiol 2009 297 R1118 1126 19675277 10.1152/ajpregu.90904.2008

[B36] Sadikot RT Han W Everhart MB Zoia O Peebles RS Jansen ED Yull FE Christman JW Blackwell TS Selective I kappa B kinase expression in airway epithelium generates neutrophilic lung inflammation J Immunol 2003 170 1091 1098 12517978 10.4049/jimmunol.170.2.1091

[B37] Everhart MB Han W Sherrill TP Arutiunov M Polosukhin VV Burke JR Sadikot RT Christman JW Yull FE Blackwell TS Duration and intensity of NF-kappaB activity determine the severity of endotoxin-induced acute lung injury J Immunol 2006 176 4995 5005 16585596 10.4049/jimmunol.176.8.4995

[B38] Sadikot RT Zeng H Yull FE Li B Cheng DS Kernodle DS Jansen ED Contag CH Segal BH Holland SM p47phox deficiency impairs NF-kappa B activation and host defense in Pseudomonas pneumonia J Immunol 2004 172 1801 1808 14734763 10.4049/jimmunol.172.3.1801

[B39] Sadikot RT Zeng H Joo M Everhart MB Sherrill TP Li B Cheng DS Yull FE Christman JW Blackwell TS Targeted immunomodulation of the NF-kappaB pathway in airway epithelium impacts host defense against Pseudomonas aeruginosa J Immunol 2006 176 4923 4930 16585588 10.4049/jimmunol.176.8.4923

[B40] Notebaert S Carlsen H Janssen D Vandenabeele P Blomhoff R Meyer E In vivo imaging of NF-kappaB activity during Escherichia coli-induced mammary gland infection Cell Microbiol 2008 10 1249 1258 10.1111/j.1462-5822.2008.01123.x 18241210

[B41] Connelly L Barham W Pigg R Saint-Jean L Sherrill T Cheng DS Chodosh LA Blackwell TS Yull FE Activation of nuclear factor kappa B in mammary epithelium promotes milk loss during mammary development and infection J Cell Physiol 2010 222 73 81 10.1002/jcp.21922 19746431 PMC2783968

[B42] Williams WM Staines NA Muller S Isenberg DA Human T cell responses to autoantibody variable region peptides Lupus 1995 4 464 471 10.1177/096120339500400608 8749569

[B43] Dayan M Segal R Sthoeger Z Waisman A Brosh N Elkayam O Eilat E Fridkin M Mozes E Immune response of SLE patients to peptides based on the complementarity determining regions of a pathogenic anti-DNA monoclonal antibody J Clin Immunol 2000 20 187 194 10.1023/A:1006685413157 10941826

[B44] van Schooten WC Devereux D Ho CH Quan J Aguilar BA Rust CJ Joint-derived T cells in rheumatoid arthritis react with self-immunoglobulin heavy chains or immunoglobulin-binding proteins that copurify with immunoglobulin Eur J Immunol 1994 24 93 98 10.1002/eji.1830240115 8020576

[B45] Holmoy T Fredriksen AB Thompson KM Hestvik AL Bogen B Vartdal F Cerebrospinal fluid T cell clones from patients with multiple sclerosis: recognition of idiotopes on monoclonal IgG secreted by autologous cerebrospinal fluid B cells Eur J Immunol 2005 35 1786 1794 10.1002/eji.200425417 15864781

[B46] Munthe LA Corthay A Os A Zangani M Bogen B Systemic autoimmune disease caused by autoreactive B cells that receive chronic help from Ig V region-specific T cells J Immunol 2005 175 2391 2400 16081810 10.4049/jimmunol.175.4.2391

[B47] Zangani M Carlsen H Kielland A Os A Hauglin H Blomhoff R Munthe LA Bogen B Tracking early autoimmune disease by bioluminescent imaging of NF-kappaB activation reveals pathology in multiple organ systems Am J Pathol 2009 174 1358 1367 10.2353/ajpath.2009.080700 19286564 PMC2671367

[B48] Izmailova ES Paz N Alencar H Chun M Schopf L Hepperle M Lane JH Harriman G Xu Y Ocain T Use of molecular imaging to quantify response to IKK-2 inhibitor treatment in murine arthritis Arthritis Rheum 2007 56 117 128 10.1002/art.22303 17195214

[B49] Kielland A Blom T Nandakumar KS Holmdahl R Blomhoff R Carlsen H In vivo imaging of reactive oxygen and nitrogen species in inflammation using the luminescent probe L-012 Free Radic Biol Med 2009 47 760 766 10.1016/j.freeradbiomed.2009.06.013 19539751

[B50] Arkan MC Hevener AL Greten FR Maeda S Li ZW Long JM Wynshaw-Boris A Poli G Olefsky J Karin M IKK-beta links inflammation to obesity-induced insulin resistance Nat Med 2005 11 191 198 10.1038/nm1185 15685170

[B51] Carlsen H Haugen F Zadelaar S Kleemann R Kooistra T Drevon CA Blomhoff R Diet-induced obesity increases NF-kappaB signaling in reporter mice Genes Nutr 2009 4 215 222 10.1007/s12263-009-0133-6 19707810 PMC2745749

[B52] Chiang SH Bazuine M Lumeng CN Geletka LM Mowers J White NM Ma JT Zhou J Qi N Westcott D The protein kinase IKKepsilon regulates energy balance in obese mice Cell 2009 138 961 975 10.1016/j.cell.2009.06.046 19737522 PMC2756060

[B53] Austenaa LM Carlsen H Ertesvag A Alexander G Blomhoff HK Blomhoff R Vitamin A status significantly alters nuclear factor-kappaB activity assessed by in vivo imaging Faseb J 2004 18 1255 1257 15180954 10.1096/fj.03-1098fje

[B54] Austenaa LM Carlsen H Hollung K Blomhoff HK Blomhoff R Retinoic acid dampens LPS-induced NF-kappaB activity: results from human monoblasts and in vivo imaging of NF-kappaB reporter mice J Nutr Biochem 2009 20 726 734 10.1016/j.jnutbio.2008.07.002 18926686

[B55] Lin AH Luo J Mondshein LH ten Dijke P Vivien D Contag CH Wyss-Coray T Global analysis of Smad2/3-dependent TGF-beta signaling in living mice reveals prominent tissue-specific responses to injury J Immunol 2005 175 547 554 15972691 10.4049/jimmunol.175.1.547

[B56] Rubtsov YP Rudensky AY TGFbeta signalling in control of T-cell-mediated self-reactivity Nat Rev Immunol 2007 7 443 453 10.1038/nri2095 17525753

[B57] Zhang N Weber A Li B Lyons R Contag PR Purchio AF West DB An inducible nitric oxide synthase-luciferase reporter system for in vivo testing of anti-inflammatory compounds in transgenic mice J Immunol 2003 170 6307 6319 12794164 10.4049/jimmunol.170.12.6307

[B58] Zhang N Ahsan MH Zhu L Sambucetti LC Purchio AF West DB Regulation of IkappaBalpha expression involves both NF-kappaB and the MAP kinase signaling pathways J Inflamm (Lond) 2005 2 10 10.1186/1476-9255-2-10 16207380 PMC1262753

[B59] Zhang N Ahsan MH Purchio AF West DB Serum amyloid A-luciferase transgenic mice: response to sepsis, acute arthritis, and contact hypersensitivity and the effects of proteasome inhibition J Immunol 2005 174 8125 8134 15944321 10.4049/jimmunol.174.12.8125

[B60] Zhang N Ahsan MH Zhu L Sambucetti LC Purchio AF West DB NF-kappaB and not the MAPK signaling pathway regulates GADD45beta expression during acute inflammation J Biol Chem 2005 280 21400 21408 10.1074/jbc.M411952200 15797874

[B61] Hyoudou K Nishikawa M Kobayashi Y Kuramoto Y Yamashita F Hashida M Analysis of in vivo nuclear factor-kappaB activation during liver inflammation in mice: prevention by catalase delivery Mol Pharmacol 2007 71 446 453 10.1124/mol.106.027169 17105872

[B62] Gross S Piwnica-Worms D Real-time imaging of ligand-induced IKK activation in intact cells and in living mice Nat Methods 2005 2 607 614 10.1038/nmeth779 16094386

[B63] Peterson JR Infanger DW Braga VA Zhang Y Sharma RV Engelhardt JF Davisson RL Longitudinal noninvasive monitoring of transcription factor activation in cardiovascular regulatory nuclei using bioluminescence imaging Physiol Genomics 2008 33 292 299 10.1152/physiolgenomics.00296.2007 18230667

[B64] Kruttwig K Brueggemann C Kaijzel E Vorhagen S Hilger T Lowik C Hoehn M Development of a Three-Dimensional In Vitro Model for Longitudinal Observation of Cell Behavior: Monitoring by Magnetic Resonance Imaging and Optical Imaging Mol Imaging Biol 2009 in press 19949979 10.1007/s11307-009-0289-x PMC2912723

[B65] Didierlaurent A Ferrero I Otten LA Dubois B Reinhardt M Carlsen H Blomhoff R Akira S Kraehenbuhl JP Sirard JC Flagellin promotes myeloid differentiation factor 88-dependent development of Th2-type response J Immunol 2004 172 6922 6930 15153511 10.4049/jimmunol.172.11.6922

[B66] Smeets RL Joosten LA Arntz OJ Bennink MB Takahashi N Carlsen H Martin MU Berg WB van den Loo FA van de Soluble interleukin-1 receptor accessory protein ameliorates collagen-induced arthritis by a different mode of action from that of interleukin-1 receptor antagonist Arthritis Rheum 2005 52 2202 2211 10.1002/art.21108 15986350

[B67] Vykhovanets EV Shukla S MacLennan GT Resnick MI Carlsen H Blomhoff R Gupta S Molecular imaging of NF-kappaB in prostate tissue after systemic administration of IL-1 beta Prostate 2008 68 34 41 10.1002/pros.20666 18004768

[B68] Ruusalepp A Yan ZQ Carlsen H Czibik G Hansson GK Moskaug JO Blomhoff R Valen G Gene deletion of NF-kappaB p105 enhances neointima formation in a mouse model of carotid artery injury Cardiovasc Drugs Ther 2006 20 103 111 10.1007/s10557-006-6755-7 16534546

[B69] Alexander G Carlsen H Blomhoff R Strong in vivo activation of NF-kappaB in mouse lenses by classic stressors Invest Ophthalmol Vis Sci 2003 44 2683 2688 10.1167/iovs.02-0829 12766073

[B70] Alexander G Carlsen H Blomhoff R Corneal NF-kappaB activity is necessary for the retention of transparency in the cornea of UV-B-exposed transgenic reporter mice Exp Eye Res 2006 82 700 709 10.1016/j.exer.2005.09.012 16289165

[B71] Didierlaurent AM Morel S Lockman L Giannini SL Bisteau M Carlsen H Kielland A Vosters O Vanderheyde N Schiavetti F AS04, an aluminum salt- and TLR4 agonist-based adjuvant system, induces a transient localized innate immune response leading to enhanced adaptive immunity J Immunol 2009 183 6186 6197 10.4049/jimmunol.0901474 19864596

[B72] Dohlen G Carlsen H Blomhoff R Thaulow E Saugstad OD Reoxygenation of hypoxic mice with 100% oxygen induces brain nuclear factor-kappa B Pediatr Res 2005 58 941 945 10.1203/01.PDR.0000182595.62545.EE 16183808

[B73] Dohlen G Odland HH Carlsen H Blomhoff R Thaulow E Saugstad OD Antioxidant activity in the newborn brain: a luciferase mouse model Neonatology 2008 93 125 131 10.1159/000107777 17785990

[B74] Ertesvag A Austenaa LM Carlsen H Blomhoff R Blomhoff HK Retinoic acid inhibits in vivo interleukin-2 gene expression and T-cell activation in mice Immunology 2009 126 514 522 10.1111/j.1365-2567.2008.02913.x 18778286 PMC2673363

[B75] Partridge J Carlsen H Enesa K Chaudhury H Zakkar M Luong L Kinderlerer A Johns M Blomhoff R Mason JC Laminar shear stress acts as a switch to regulate divergent functions of NF-kappaB in endothelial cells Faseb J 2007 21 3553 3561 10.1096/fj.06-8059com 17557931

[B76] Tillmanns J Carlsen H Blomhoff R Valen G Calvillo L Ertl G Bauersachs J Frantz S Caught in the act: in vivo molecular imaging of the transcription factor NF-kappaB after myocardial infarction Biochem Biophys Res Commun 2006 342 773 774 10.1016/j.bbrc.2006.02.024 16497270

[B77] Kielland A Camassa LA Munthe LA Amiry-Moghaddam M Blomhoff R Carlsen H NF-kB is upregulated in endothelial cells in brain following ischemia Manuscript 2010 in press

[B78] Blackwell TS Yull FE Chen CL Venkatakrishnan A Blackwell TR Hicks DJ Lancaster LH Christman JW Kerr LD Use of genetically altered mice to investigate the role of nuclear factor-kappa B activation and cytokine gene expression in sepsis-induced ARDS Chest 1999 116 73S 74S 10.1378/chest.116.suppl_1.73S 10424600

[B79] Blackwell TS Yull FE Chen CL Venkatakrishnan A Blackwell TR Hicks DJ Lancaster LH Christman JW Kerr LD Multiorgan nuclear factor kappa B activation in a transgenic mouse model of systemic inflammation Am J Respir Crit Care Med 2000 162 1095 1101 10988136 10.1164/ajrccm.162.3.9906129

[B80] Sadikot RT Wudel LJ Jansen DE Debelak JP Yull FE Christman JW Blackwell TS Chapman WC Hepatic cryoablation-induced multisystem injury: bioluminescent detection of NF-kappaB activation in a transgenic mouse model J Gastrointest Surg 2002 6 264 270 10.1016/S1091-255X(01)00064-6 11992813

[B81] Stathopoulos GT Sherrill TP Han W Sadikot RT Yull FE Blackwell TS Fingleton B Host nuclear factor-kappaB activation potentiates lung cancer metastasis Mol Cancer Res 2008 6 364 371 10.1158/1541-7786.MCR-07-0309 18337446

[B82] Gray KD Simovic MO Chapman WC Blackwell TS Christman JW Washington MK Yull FE Jaffal N Jansen ED Gautman S Stain SC Systemic nf-kappaB activation in a transgenic mouse model of acute pancreatitis J Surg Res 2003 110 310 314 10.1016/S0022-4804(03)00024-6 12697416

[B83] Jimi E Phillips RJ Rincon M Voll R Karasuyama H Flavell R Ghosh S Activation of NF-kappaB promotes the transition of large, CD43+ pre-B cells to small, CD43- pre-B cells Int Immunol 2005 17 815 825 10.1093/intimm/dxh263 15908447

[B84] Millet I Phillips RJ Sherwin RS Ghosh S Voll RE Flavell RA Vignery A Rincon M Inhibition of NF-kappaB activity and enhancement of apoptosis by the neuropeptide calcitonin gene-related peptide J Biol Chem 2000 275 15114 15121 10.1074/jbc.275.20.15114 10809748

[B85] Voll RE Jimi E Phillips RJ Barber DF Rincon M Hayday AC Flavell RA Ghosh S NF-kappa B activation by the pre-T cell receptor serves as a selective survival signal in T lymphocyte development Immunity 2000 13 677 689 10.1016/S1074-7613(00)00067-4 11114380

[B86] Hubbard AK Timblin CR Shukla A Rincon M Mossman BT Activation of NF-kappaB-dependent gene expression by silica in lungs of luciferase reporter mice Am J Physiol Lung Cell Mol Physiol 2002 282 L968 975 11943661 10.1152/ajplung.00327.2001

[B87] Ho TY Chen YS Hsiang CY Noninvasive nuclear factor-kappaB bioluminescence imaging for the assessment of host-biomaterial interaction in transgenic mice Biomaterials 2007 28 4370 4377 10.1016/j.biomaterials.2007.07.005 17645941

[B88] Magness ST Jijon H Van Houten Fisher N Sharpless NE Brenner DA Jobin C In vivo pattern of lipopolysaccharide and anti-CD3-induced NF-kappa B activation using a novel gene-targeted enhanced GFP reporter gene mouse J Immunol 2004 173 1561 1570 15265883 10.4049/jimmunol.173.3.1561

[B89] Ando T Hatsushika K Wako M Ohba T Koyama K Ohnuma Y Katoh R Ogawa H Okumura K Luo J Orally administered TGF-beta is biologically active in the intestinal mucosa and enhances oral tolerance J Allergy Clin Immunol 2007 120 916 923 10.1016/j.jaci.2007.05.023 17606291

[B90] Luo J Lin AH Masliah E Wyss-Coray T Bioluminescence imaging of Smad signaling in living mice shows correlation with excitotoxic neurodegeneration Proc Natl Acad Sci USA 2006 103 18326 18331 10.1073/pnas.0605077103 17110447 PMC1838750

[B91] Ishikawa TO Herschman HR Tumor formation in a mouse model of colitis associated colon cancer does not require COX-1 or COX-2 expression Carcinogenesis 2010 20061361 10.1093/carcin/bgq002 PMC2847091

[B92] Ishikawa TO Jain N Herschman HR Feedback regulation of cyclooxygenase-2 transcription ex vivo and in vivo Biochem Biophys Res Commun 2009 378 534 538 10.1016/j.bbrc.2008.11.099 19061862 PMC2637547

[B93] Ishikawa TO Jain NK Taketo MM Herschman HR Imaging cyclooxygenase-2 (Cox-2) gene expression in living animals with a luciferase knock-in reporter gene Mol Imaging Biol 2006 8 171 187 10.1007/s11307-006-0034-7 16557423

